# Age-related attenuation of cortical synaptic tagging in the ACC is rescued by BDNF or a TrkB receptor agonist in both sex of mice

**DOI:** 10.1186/s13041-022-00992-x

**Published:** 2023-01-06

**Authors:** Si-Bo Zhou, Man Xue, Weiqi Liu, Yu-Xin Chen, Qi-Yu Chen, Jing-Shan Lu, Jinjun Wang, Keqiang Ye, Xu-Hui Li, Min Zhuo

**Affiliations:** 1 Center for Neuron and Disease, Frontier Institutes of Science and Technology, Xi’an Jiaotong University, Xi’an, China; 2 Institute of Brain Research, Qingdao International Academician Park, Qingdao, Shandong China; 3 Institute of Artificial Intelligence and Robotics, Xi’an Jiaotong University, Xi’an, China; 4 CAS Key Laboratory of Brain Connectome and Manipulation, Interdisciplinary Center for Brain Information, The Brain Cognition and Brain Disease Institute, Shenzhen-Hong Kong Institute of Brain Science-Shenzhen Fundamental Research Institutions, Shenzhen Institute of Advanced Technology, Chinese Academy of Sciences Shenzhen Institute of Advanced Technology, Shenzhen, China; 5 Pathology and Laboratory Medicine, Emory University School of Medicine, Atlanta, GA USA; 6 Faculty of Life and Health Sciences, Brain Cognition and Brain Disease Institute, Shenzhen Institute of Advanced Technology, Chinese Academy of Sciences, Shenzhen, China; 7 Oujiang Laboratory, Zhejiang Lab for Regenerative Medicine, Vision and Brain Health, Wenzhou, 325000 Zhejiang China; 8 Department of Physiology, Faculty of Medicine, University of Toronto, Medical Science Building, 1 King’s College Circle, Toronto, ON M5S 1A8 Canada

**Keywords:** Synaptic tagging, LTP, BDNF, R13, ACC, Middle-aged mice

## Abstract

Long-term potentiation (LTP) is a key cellular mechanism for learning and memory, and recent studies in the hippocampus found that LTP was impaired in aged animals. Previous studies of cortical LTP have focused primarily on the homosynaptic plasticity in adult mice, while fewer studies have looked at heterosynaptic plasticity—such as synaptic tagging in aged mice. In the present study, we investigated synaptic tagging in adult and middle-aged mice's anterior cingulate cortex (ACC) using the 64-channel multielectrode dish (MED64) recording system. We found that synaptic tagging was impaired in the ACC of middle-aged male mice as compared to adult mice. Both the network late-phase LTP (L-LTP) and the recruitment of inactive responses were reduced in the ACC of middle-aged male mice. Similar results were found in female middle-aged mice, indicating that there is no gender difference. Furthermore, bath application of brain-derived neurotrophic factor (BDNF) or systemic treatment with newly developed TrkB receptor agonists R13, was shown to rescue both synaptic tagging, and L-LTP, in middle-aged mice. To determine the distribution of synaptic LTP within the ACC, a new visualization method was developed to map the Spatio-temporal variation of LTP in the ACC. Our results provide strong evidence that cortical potentiation and synaptic tagging show an age-dependent reduction, and point to the TrkB receptor as a potential drug target for the treatment of memory decline.

## Introduction

Long-term potentiation (LTP), an activity-dependent long-lasting increase of synaptic efficacy caused by high-frequency stimulation or theta burst stimulation (TBS), has been established as a cellular model of memory in different regions of the brain, including the hippocampus, prefrontal cortex, and the anterior cingulate cortex (ACC) [1–3]. LTP has at least two distinct temporal phases: protein synthesis-independent early-phase LTP (E-LTP), and protein synthesis-dependent late-phase LTP (L-LTP) [4–6]. Furthermore, it has been reported that E-LTP and L-LTP can interact with each other in a 'synaptic tagging-like manner. Weak tetanus-inducing E-LTP sets a “tag”, which can capture the plasticity-related proteins (PRPs) synthesized following the strong tetanus-inducing L-LTP [7–9]. A weak stimulus can induce L-LTP if it is preceded or followed by strong tetanus given to a separate, independent pathway that converges into the same neuronal population. This finding has been subsequently repeated and extensively investigated [10].

Synaptic tagging is not just limited to the hippocampus. In the ACC, a cortical region that is important for pain perception and emotional memory process [11–17], synaptic tagging has also been reported. Similar to our findings in the hippocampus, our previous studies reported that weak TBS can also induce heterosynaptic synaptic tagging in the ACC of adult mice, which depends on a certain time window and the synthesis of new proteins[18]. Functionally, several lines of evidence suggest that synaptic tagging may contribute to memory allocation and storage [19–21]. It may also provide a synaptic mechanism for emotional tagging [22]. For example, Liu et al. previously reported that tail amputation-induced peripheral injury caused a loss of hetero-synaptic L-LTP in the ACC [18]. Vecsey et al. found that sleep deprivation impaired synaptic tagging in the mouse hippocampus [23]. Most of the previous synaptic tagging studies have focused primarily on adult mice—it is unclear to this point whether synaptic tagging is affected by aging.

Age-related synaptic LTP and memory impairment has been reported in the hippocampus and hippocampus-dependent behavioral tests [24–26]. Interestingly, age-related impairment of behavioral tagging and synaptic tagging has also been reported in the hippocampus [27, 28]. Wong et al. (2021) reported that synaptic tagging was attenuated in the hippocampal region of middle-aged mice [27]. There is no report of age-related changes in synaptic tagging in the ACC. Our recent studies using animal models of amputation found that synaptic tagging in the ACC was either reduced or abolished in ACC slices after tail amputation [18], suggesting that cortical synaptic tagging is plastic, and may possibly be affected by either peripheral injury or aging.

In the present study, we employed the MED64 recording system to investigate synaptic tagging in the ACC of both adult and middle-aged mice. Both male and female mice were used. We found that synaptic tagging in the ACC was significantly reduced in middle-aged animals as compared to adults. Cumulative data has demonstrated that brain-derived neurotrophic factor (BDNF) is crucially involved in synaptic plasticity in the adult brain [29]. Furthermore, by using BDNF—or selective trkB receptor agonist R13—we were able to reverse the loss of synaptic tagging in middle-aged animals. Finally, we developed a novel method to better visualize the Spatio-temporal signals of fEPSP signals and multiple LTP responses within the ACC circuit from low-resolution MED64 inputs.

## Materials and methods

### Animals

For the animal groups, we divided them into two major groups by age: adult mice (6–8 weeks), and middle-aged mice (50–60 weeks). All mice were done on male and female C57BL/6 mice purchased from the Experimental Animal Center of Xi’an Jiaotong University (6–8 weeks) and Charles River Laboratories in Beijing (50–60 weeks). All mice were randomly housed by three to four per cage under standard laboratory conditions (12 h light/12 h dark, temperature 22–26 °C, air humidity 55–60%). Food and water were available ad-lib. All research protocols performed in this experiment were approved by the Ethics Committee of Xi’an Jiaotong University.

### Preparation of the multi-electrode array

There is an array of 64 square planar microelectrodes (50 × 50 µm/each) arranged in an 8 × 8 pattern in the MED64 probe (P515A, chamber depth 10 mm, Alpha MED Scientific, Japan), with an interpolar distance of 150 μm. Since the surface of the MED64 probe is relatively hydrophobic, in order to attach the slice to the MED64 probe well, the new MED64 probe received hydrophilic treatment. Before experiments, we treated the surface of the MED64 probe with 0.1% polyethyleneimine (Sigma, St. Louis, MO; P-3143) in 25 mmol/L borate buffer (pH 8.4) overnight at room temperature. Then we used sterile distilled water to flush the probe surface three times to remove any harmful substances that may affect the activity of brain slices [30, 31].

### Brain slice preparation

The general procedures for making the ACC slices were similar to that in our previous study [30, 31]. Acute coronal brain slices (300 μm) containing ACC were prepared from C57BL/6 mice. In brief, we anesthetized C57BL/6 mice with 1–2% isoflurane and sacrificed them by decapitation. The entire brain was quickly removed from the skull and submerged in an ice-cold oxygenated (equilibrated with 95% O_2_ and 5% CO_2_) cutting solution containing (in mM) 252 sucrose, 2.5 KCl, 6 MgSO_4_, 0.5 CaCl_2_, 25 NaHCO_3_, 1.2 NaH_2_PO_4_, and 10 glucose, pH 7.3 to 7.4 for a short time. After a brief cooling, the brain was trimmed, and the remaining brain block was glued onto the ice-cold stage of a vibrating tissue slicer (Leica, VT1200S). In this way, the brain coronal brain slices (300 μm) containing ACC were obtained, and the slices were then transferred to a submerged recovery chamber with oxygenated (95% O_2_ and 5% CO_2_) artificial cerebrospinal fluid (ACSF) containing (in mM) NaCl 124, KCl 2.5, CaCl_2_ 2, MgSO_4_ 2, NaHCO_3_ 25, NaH_2_PO_4_ 1 and glucose 10, pH 7.3–7.4 at room temperature for at least 1.5 h. This ACSF was used throughout the experiment, including the recording stage.

### Field potential recording

**Fig. 1:**
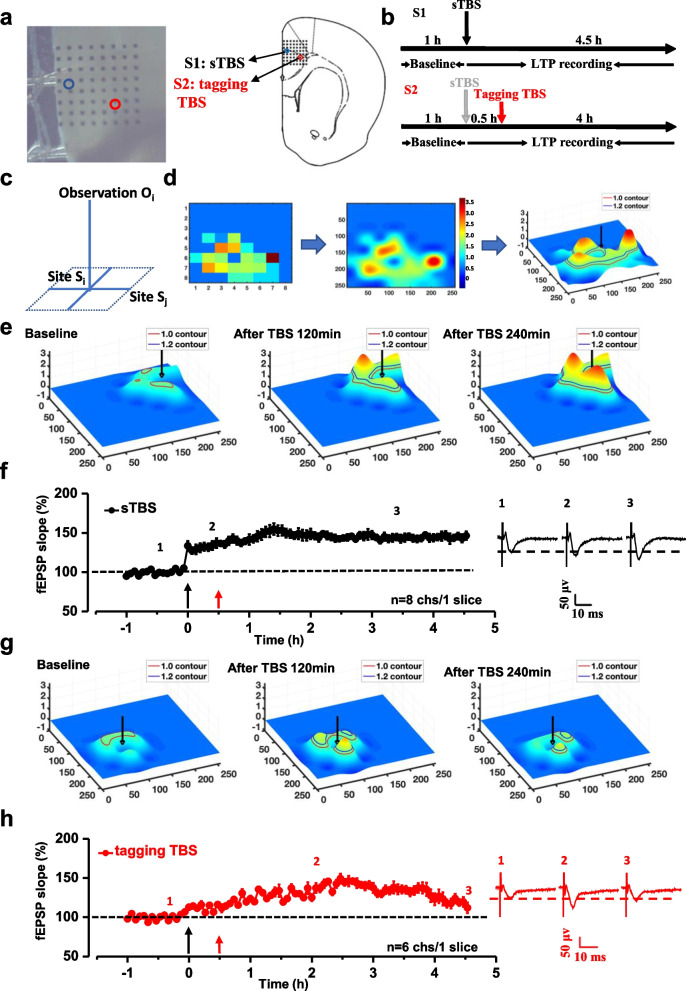
Synaptic tagging was induced in the ACC by tagging TBS in middle-aged male mice. Tagging was recorded from a slice of a male mouse by extracellular field potential recording. **a** Microscopy photograph showing the relative location of ACC slice and MED64 probe and the arrangement of the microelectrodes (electrode size 50 × 50 μm, the interpolar distance of electrodes 150 μm). Schematic diagram of stimulation site (S1: blue circle for the strong TBS; S2: red circle for the weak TBS) of microelectrodes in the ACC. Spatial distribution of extracellular field potential induced by strong TBS on channel 26 (marked as a blue circle) in layers II and weak TBS on channel 46 (marked as a red circle) in layers V of the ACC. **b** Schematic diagram of the recording procedure for sites S1 and S2. **c** The Markov Random Field model was introduced to smooth and visualize the low-resolution 8 × 8 MED64 data. The graph model consists of the observation at site i (O_i_), the state at site i (S_i_), and the state at a neighboring site j (S_j_). **d** As a generative process, the value of O_i_ is generated from S_i_, while the value of S_i_ also depends on its 4-connection neighborhood denoted as S_j_. The original 8 × 8 MED64 record for a single time frame. Then, a 256 × 256 projection of the original MED64 data that is both spatially and temporally smoother with a color bar. Finally, the temporal evolving of the 3D surface of the projected 256 × 256 MED64 recording. **e** The temporal evolving of the 3D surface on the site S1 in the middle-aged mice. **f** Summarized plot of the time-varying fEPSP slope in all activated channels for site S1 from 1 slice of a middle-aged male mouse. **g** The temporal evolving of the 3D surface on site S2 of the middle-aged mice. **h** Summarized plot of time-varying fEPSP slopes in all activated channels for tagging TBS protocol from the 1 slice

A commercial 64-channel recording system (MED64, Panasonic Alpha-Med Sciences, Japan) was used to record extracellular field potential in ACC in male and female C57BL/6 mice. After incubation, one slice containing ACC was transferred to the prepared recording probe. The ACC part was placed on the MED64 probe’s electrodes, and the 64 electrodes were covered by different layers of the ACC. We can easily choose the superficial layers (layers II-III) or deep layers (layers V-VI) as the stimuli site. Once the slice was settled, a mesh and an anchor (Warner Instruments, Harvard) were carefully positioned to ensure the stability of the slice during recording. The slice was perfused continuously with oxygenated (95% O_2_ and 5% CO_2_) ACSF at 26–28 ℃ and maintained at a 2–3 ml/min flow rate with the aid of a peristaltic pump (Minipuls 3, Gilson) throughout the experiments. Figure 1a shows a micrograph of an ACC slice placed on a MED64 probe. Before the experiment, the slices were kept in the probe for at least 1 h.

For test stimulation, constant current pulses (0.2 ms in duration) generated by a data acquisition software (Mobius, Panasonic Alpha-Med Sciences) were applied to either deep layers (layer V-VI, for tagging TBS input) and/or superficial layers (layer II-III, for strong TBS input) of the ACC slice. Bipolar constant current pulse stimulation (6–10 µA, 0.2 ms) was applied to the stimulation site, and the intensity was adjusted so that we could evoke a half-maximum field excitatory postsynaptic potential (fEPSP) in the channel nearest to the stimulation site. The channel with fEPSP was regarded as an activated channel, and its fEPSP response was sampled every 2 min and averaged every 4 min. The ‘slope’ parameter represented the average slope of each fEPSP recorded by the activated channel. Stable baseline responses (variation in the baseline response of a single channel is < 5% and the number of channels with unstable baseline responses was ≤ 10% of the total number of active channels) were first recorded for 60 min. Then, a strong TBS (five trains of bursts with four pulses at 100 Hz at 200 ms intervals; repeated five times at intervals of 10 s, 4 × 5×5) with the same intensity as the baseline stimulation was applied to the same stimulation channel to induce LTP. After a half hour of strong TBS, a tagging TBS (five trains of bursts with four pulses at 100 Hz at 200 ms intervals, 4 × 5×1) with the same intensity as the baseline stimulation in the deep layer was applied to the same stimulation channel to induce synaptic tagging. After the induction of LTP or/and tagging LTP, the fEPSP responses were continued recording for another 4–4.5 h. The fEPSPs of sites S1 and S2 will be recorded in turn, with an interval of one minute.

We also applied BDNF to the brain slices of middle-aged mice. We added 1 µg BDNF to 20 ml oxygenated ACSF to perfuse the brain slice. The ACSF with BDNF was applied for 1 h, from 15 min after strong TBS to 45 min after tagging TBS. Then the BDNF was washed out. Synaptic tagging in R13 oral gavage of middle-aged mice was also studied. After R13 oral administration (concentration: 43.6 mg/kg/d) for 15 days, we performed the same field potential recording protocol as above mentioned. R13 was dissolved in pure DMSO, then suspended in 0.5% methylcellulose at a final concentration of 5% DMSO/0.5% methylcellulose.

### Western blot

After the fEPSPs recording, the ACC was collected from the brain slices on ice in cold PBS and homogenized in lysis buffer (10 mM Tris–HCl (pH 7.4), 2 mM EDTA, 1% SDS including a protease inhibitor cocktail). Samples were then centrifuged (12,000*g*, 20 min, 4 °C) for supernatant. Western blot was performed as previously described [32]. Sample protein concentrations were quantified using Bradford assay (Beyotime), and electrophoresis of equal amounts of protein (30 μg) was performed on 7.5% SDS–polyacrylamide gel. Separated proteins were transferred to polyvinylidene fluoride (PVDF) membranes, followed by blocking with 5% skim milk in TBS-T (Tris-buffered saline with Triton X-100) at room temperature for 1 h, and were then probed with primary antibody: anti-Trk B receptor (1:1000, rabbit polyclonal, Santa Cruz Biotechnology), anti-CREB (1:1000, rabbit polyclonal, Abcam) and anti-GAPDH (1:10,000, rabbit polyclonal, Abcam) at 4 °C overnight. The membranes were incubated with horseradish peroxidase-coupled anti-rabbit/mouse lgG secondary antibody diluted at 1:5000 (Millipore) for 1 h, followed by enhanced chemiluminescence detection of the proteins with Enhanced chemiluminescence, ECL (GE Healthcare). ImageJ software (National Institute of Health) was used to assess the intensity of immunoblots.

### A novel fEPSP signal modeling and visualization system

We consider the recorded $8\times 8$ Med64 data as a partially observed Markov Random Field [33]. Specifically, we assume that the original data is from a larger field with $\mathrm{N}\times \mathrm{ N}$ sites. Typically, $\mathrm{N}\ge 8$ but technically $\mathrm{N}$ can be any integer value greater than zero. We define the state space to be continuous in ${[\mathrm{m}}_{\mathrm{min}}, {\mathrm{m}}_{\mathrm{max}}]$, and for visualization purpose, we stretch the range to be discrete within [$0, 255]$. Among the $\mathrm{N}\times \mathrm{ N}$ sites, $8\times 8$ state has observations$\mathrm{O}=\left\{{\mathrm{o}}_{0},\dots ,{\mathrm{o}}_{63}\right\}$. Therefore, the MRF has the following structure as shown in Fig. 1c.

We aim to estimate the state value of all the sites $X=\{{x}_{0},\dots ,{x}_{N\times N}\}$ with the *Maximum *a Posteriori* (MAP)* criterion based on the partial observation and the interdependency between sites. The MAP estimation of the MRF strives to minimize the following loss,

1 $$\Phi \left(X\right){=\Phi }_{\mathrm{unary}}\left(X\right)+{\Phi }_{\mathrm{pairwise}}\left(X\right)$$

where the unary term considers the consistency from observation and is defined by

2 $${\Phi }_{\mathrm{unary}}\left(X\right)={\sum }_{i=0}^{8\times 8}{{(o}_{i}-{x}_{i})}^{2}$$

and the pairwise term considers the interdependency between sites and is defined by

3 $${\Phi }_{\mathrm{pairwise}}\left(X\right)={\sum }_{i=0}^{N\times N}\sum_{j\in \mathcal{N}\left(i\right)}{{{a}_{i,j}(x}_{i}-{x}_{j})}^{2}$$

Specifically, the smoothness weight ${a}_{i,j}$ between site $i$ and $j$ is defined using the Lanczos [34] filter by

4 $${\mathrm{a}}_{\mathrm{i},\mathrm{j}}=\frac{\alpha sin\left(\pi (i-j)\right)sin\left(\pi (i-j)/\alpha \right)}{{\pi }^{2}{(i-j)}^{2}}$$

and $\mathcal{N}\left(i\right)$ denotes a neighborhood of site $i$ and is controlled by $\alpha$. In our implementation, we set $\alpha =3$ and hence $\mathcal{N}\left(i\right)$ includes sites from within a $5\times 5$ grid region centering at $i$.

The following figures (Fig. 1d) illustrate the original $8\times 8$ Med64 data, the obtained $256\times 256$ estimation, and visualization of the $256\times 256$ surface with some additional information.

Since we have a continuous recording of the MED64 signal, we could actually further consider a more complete spatio-temporal relationship between sites to get a more stable, smooth and possibly more accurate estimation of the site value. Mathematically this is achievable by defining a 3D neighborhood system in addition to Eq. (3), we have

5 $${\Phi }_{\mathrm{pairwise}}\left(X\right)={\sum }_{i=0}^{N\times N}\sum_{t\in {\mathcal{N}}^{T}\left(i\right)}\sum_{j\in {\mathcal{N}}^{S}\left(i\right)}{{{a}_{i,j,t}(x}_{i,t}-{x}_{j,t})}^{2}$$

Specifically, the smoothness weight ${a}_{i,j,t}$ between site $i$ and $j$ spanning over $t$ frame is defined as

6 $${\mathrm{a}}_{\mathrm{i},\mathrm{j},\mathrm{t}}={\mathrm{a}}_{\mathrm{i},\mathrm{j}}{\mathrm{a}}_{\mathrm{t}}$$

where ${\mathrm{a}}_{\mathrm{i},\mathrm{j}}$ is calculated by Eq. 4, while $t$ considers the temporal distance between two sites as

7 $${\mathrm{a}}_{\mathrm{t}}=\frac{\beta -t}{\beta }$$

where $\beta$ controls the size of the temporal neighborhood, and in our implementation, we set $\beta =3$. Now, compared to the spatial case, in spatio-temporal case our $\mathcal{N}\left(i\right)$ includes sites from within a $5\times 5\times 5$ grid region centering at $i$.

### Statistical analysis

The data, either in $8\times 8$ or more generally as $\mathrm{N}\times \mathrm{ N}$ channels, is presented by channel-wise means ± SEM. Statistical comparisons between the two groups were performed using Student’s t-test to identify significant differences. In all cases, *p < 0.05 was considered statistically significant. All statistical analysis was done using SPSS Statistics.

## Results

### Impaired synaptic tagging LTP observed in the ACC of middle-aged mice

We employed a 64-channel field potential recording system and analyzed the induction probability and properties of synaptic tagging in vitro ACC slices from middle-aged male mice (50–60 weeks). Two different channels were stimulated in different layers of the ACC (S1: superficial layer; S2: deep layer). Simultaneously, the evoked multi-channel fEPSP around the stimulation sites were recorded (Fig. 1a, b). Specifically, we first applied a strong TBS (4 × 5×5) to site S1, and 30 min later, a weak TBS (tagging TBS, 4 × 5×1) was delivered to site S2. We found that normal LTP can be induced by strong TBS in middle-aged mice (Fig. 1f). There are 2 channels showing E-LTP and 6 channels showing L-LTP in 8 activity channels around site S1. However, the synaptic tagging LTP induced by weak TBS was impaired in middle-aged mice, although there were 2 channels showing tagging-like response, while the other 4 activated channels only showed the E-LTP on site S2 (Fig. 1h).

A novel fEPSP signal modeling and visualization system was developed to monitor the Spatio-temporal properties tagging LTP. We assumed that the 8 × 8 observations from the MED64 system, corresponding to the 63 recording channels plus the 64^th^ channel as the stimulation input, are only a sparse observation of the complete fEPSP signal field residing in latent state space. Such latent state space could then project into another observation space with N × N resolution where N is usually greater than 8 and was set to 256 in our experiments. In this way, the originally recorded MED64 values of fEPSP slopes were reconstructed into high-density 3D signal sequences (Fig. 1d) As shown in Fig. 1e and g, the peak intensity and spatial distribution of the fEPSP slopes were significantly increased at 120 min and 240 min after strong TBS (Fig. 1e). However, the peak intensity and spatial distribution ware slight increased at 120 min after tagging TBS in middle-aged mice (Fig. 1g). These 3D maps clearly showed that the synaptic tagging was impaired in the ACC of middle-aged mice.

**Fig. 2:**
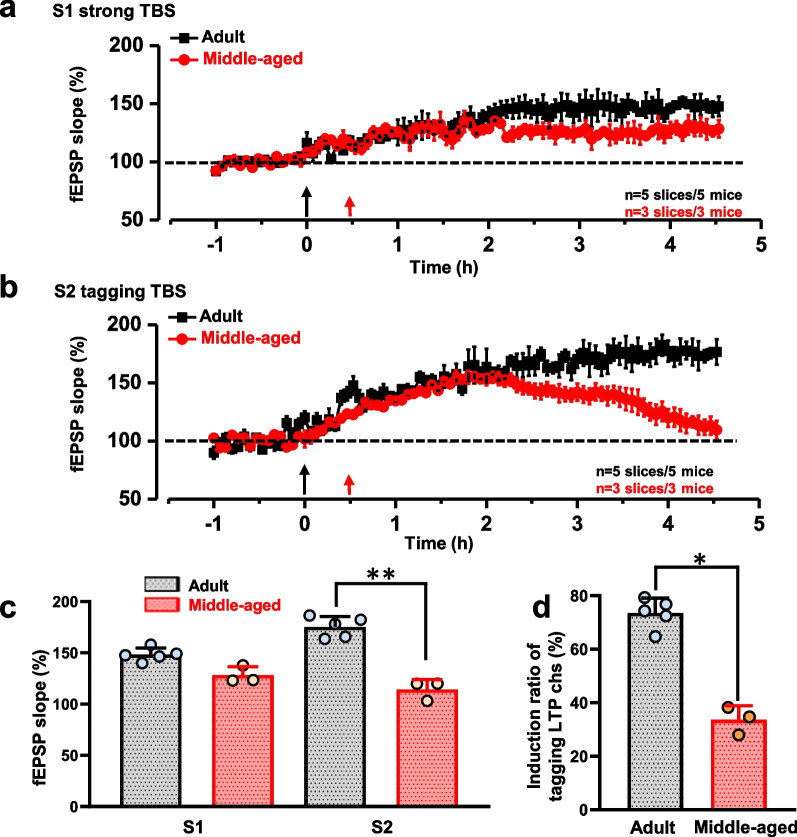
Synaptic tagging was observed in the ACC at different ages in male mice. Summarized plot of the fEPSP slope demonstrates that both strong TBS at site S1 and tagging TBS at site S2. Either adult male mice (n = 5 slices/5 mice) or middle-aged male mice (n = 3 slices/3 mice) could induce L-LTP on site S1 (**a**). The tagging-like response could be induced in adult male mice (n = 5 slices/5 mice) but not in middle-aged male mice on site S2 (n = 3 slices/3 mice) (**b**). The fEPSP slope in the last 30 min on site S1 and site S2 in each group (**c**). Percentage of activated channels that has tagging induced by tagging TBS (**d**) (**p < 0.01, Student’s *t*-test)

Next, we compared synaptic tagging response in adult mice (6–8 weeks) and middle-aged (50–60 weeks) mice. At site S1, strong TBS induced late-phase LTP (L-LTP) in all 5 adult mice, but only 3 mice showed L-LTP in 5 recorded middle-aged mice (Fig. 2a). The fEPSP slopes of the last 30 min were 148.01 ± 5.79% in adult mice and 128.04 ± 6.88% in middle-aged mice (Fig. 2c). At site S2, most channels showed tagging-like response after tagging TBS in all 5 adult mice. However, in middle-aged mice, only 3 mice out of 5 mice showed 1 or 2 tagging L-LTP, and most activated channels showed the E-LTP (Fig. 2b). The fEPSP slopes were 175.20 ± 8.92% in adult mice and 114.35 ± 7.75% in middle-aged mice (t = 42.54, **p < 0.01, n = 5 mice for each group). The conclusion could also be supported by analyzing the percentage of channels with tagging-like responses over all activated channels. The ration of the induction of tagging LTP was significantly higher in the adult group (73.33 ± 4.67%) than in the middle-aged group (33.33 ± 4.24%, t = 6.446, *p = 0.023 < 0.05, Fig. 2d).

### Less recruitment of inactive responses after synaptic tagging in middle-aged male mice

**Fig. 3:**
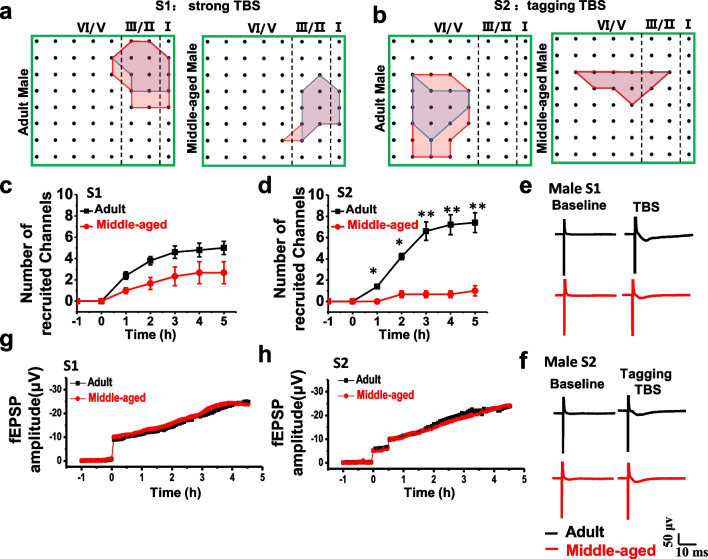
New responses were recruited after synaptic tagging in the male mice ACC. Sample slices showed the distribution of the basal activated channels (blue) and the TBS-recruited (**a**) or tagging TBS (**b**) recruited channels (red) in adult and middle-aged male mice. The temporal changes of the number (**c**, **d**) and amplitude (**g**, **h**) of the recruited fEPSPs on sites S1 and S2. (*p < 0.05, **p < 0.01, n = 5 mice for each group, Student’s *t*-test.). Samples showed the recruited responses induced by strong TBS and tagging TBS in adult and middle-aged male mice (**e**, **f**)

Previous studies have shown that the strong TBS-induced L-LTP could recruit newly-activated channels in the ACC [31, 35]. Consistently, we also found that there was clear enlargement of the response areas surrounding the strong TBS at site S1 both in adult mice and middle-aged mice (Fig. 3a). After tagging TBS on site S2, the enlargement of response areas can be observed in adult mice, but not in middle-aged mice (Fig. 3b). In adult mice, the number of recruited channels both on sites S1 and S2 gradually increased after TBS induction (Fig. 3c, d, n= 5 mice). However, in middle-aged mice, the tagging TBS on site S2 failed to increase many recruited channels. The recruited channels around site S2 are less than those in adult mice. (t_1h_ = 2.473, t_2h_ = 4.750, t_3h_ = 10.633, t_4h_ = 18.174, t_5h_ = 22.136, *p < 0.05, **p < 0.01, n = 5 mice).

The time courses of the changed fEPSP slope in the recruited channels were further shown in Fig. 3g, h. In these recruited channels, strong TBS-induced fEPSPs on site S1 were gradually potentiated, and the amplitude finally became as large as 24.79 ± 0.81 μV in adult mice and 23.85 ± 0.79 μV in middle-aged mice at 4.5 h after TBS induction (Fig. 3g). It is worth noting that the amplitudes of fEPSPs in tagging-TBS recruited channels were no significant difference in adult mice (23.90 ± 0.87 μV) and in middle-aged mice (24.09 ± 0.81 μV) when the channels occurred. (Fig. 3h).

### Synaptic tagging LTP in middle-aged female mice

**Fig. 4:**
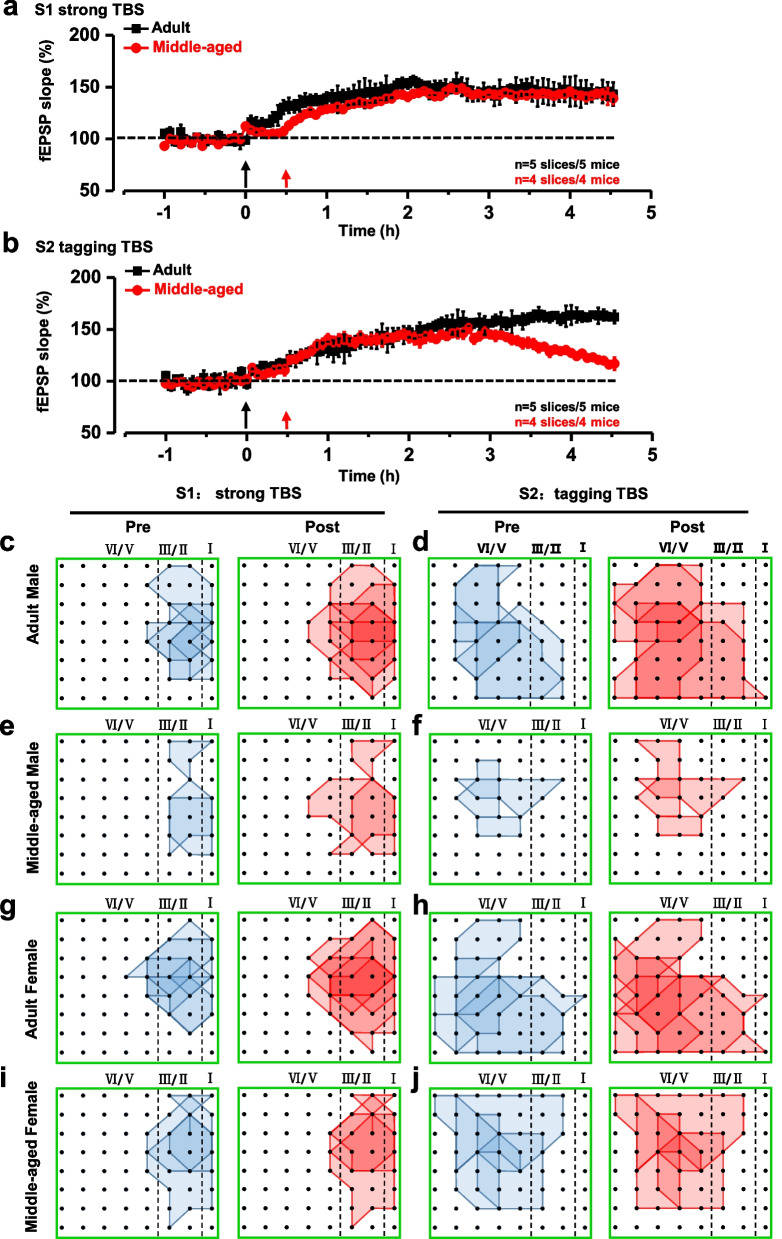
Synaptic tagging occurred in female mice. Summarized plot of the fEPSPs slope demonstrates that both strong TBS at site S1 and tagging TBS at site S2. Either adult female mice (n = 5 slices/5 mice) or middle-aged female mice (n = 4 slices/4 mice) could induce L-LTP on site S1 (**a**). Tagging-like response could be induced in adult female mice (n = 5 slices/5 mice) but not in middle-aged female mice on site S2 (n = 3 slices/3 mice) (**b**). Polygonal diagrams of channel data in each group (4 groups, n = 5) pooled from slices with the tagging-like response. **c**, **e**, **g**, **i** The channels were activated in the baseline (blue, pre) and at 4.5 h after strong TBS of the site S1 (red, post) in the slice. Black dots represent the 64 channels in the MED64 system. Vertical dashed lines indicate the layers in the ACC slice. **d**, **f**, **h**, **j** Polygonal diagrams of the channels of pool data that were activated in the baseline state (blue, pre) and at 4 h after tagging TBS (red, post) at site S2. Delivery of strong TBS resulted in an enlargement of the activation area in all groups, while tagging TBS recruit less or no new synaptic responses in the ACC in adult and aged groups

We further tested the synaptic tagging LTP in middle-aged female mice. Similar results were obtained in middle-aged female mice and male mice (Fig. 4). A summarized plot of the fEPSP slope showed that both strong TBS at the site S1 and tagging TBS at the site S2 can induce L-LTP in adult female mice (n = 5 mice). However, in the middle-aged female mice, there was no tagging LTP, and the L-LTP occurred only in site S1 in 4 slices from 5 mice (Fig. 4a, b). These results suggest that there is no gender difference for synaptic tagging LTP in middle-aged mice.

Next, we showed the spatial distribution of the active responses in the ACC before and after strong TBS and tagging TBS application across both male and female mice. The distribution of all observed activated channels was displayed by a polygonal diagram on a grid representing the channels (Fig. 4c–j).

In adult male mice, there were 55 channels exhibited clear synaptic responses from 5 slices (5 mice) at the baseline, and 26 new channels were recruited at 4.5 h after strong TBS in site S1 (Fig. 4c). Meanwhile, in middle-aged male mice, only 28 channels exhibited clear synaptic responses from 4 slices (4 mice) at the baseline, and 8 new channels were recruited at 4.5 h after strong TBS in site S1 (Fig. 4e). For tagging LTP, in adult male mice, 53 channels exhibited clear synaptic responses from 5 slices at the baseline, and 26 new channels were recruited at 4 h after tagging TBS in site S2 (Fig. 4d). In middle-aged male mice, 26 channels exhibited clear synaptic responses from 4 slices at the baseline, and only 3 new channels were recruited at 4 h after tagging TBS site S2 (Fig. 4f).

In adult female mice, 60 channels exhibited clear synaptic responses from 5 slices in 5 mice at the baseline, and 22 new channels were recruited at 4.5 h after strong TBS in site S1 (Fig. 4g). In middle-aged female mice, only 47 channels exhibited clear synaptic responses in 4 slices from 4 mice at the baseline, and 4 new channels were recruited at 4.5 h after strong TBS in site S1 (Fig. 4i). For the site S2, in adult female mice, 93 channels exhibited clear synaptic responses from 5 slices in 5 mice at the baseline, and 20 new channels were recruited at 4 h after tagging TBS (Fig. 4h). In the middle-aged female mice, 72 channels exhibited clear synaptic responses from 4 slices in 4 mice at the baseline, and only 1 new channel was recruited at 4 h after tagging TBS (Fig. 4j). Taken together, these results suggest tagging TBS can significantly enhance the spatial distribution of active responses in the adult mice, but failed to change spatial distribution of active responses in middle-aged mice.

### Age difference of weak TBS-induced L-LTP within ACC

In the above results, it was observed that synaptic tagging induced by tagging TBS (weak TBS) was more easily found in younger mice. Therefore, we wonder whether L-LTP could be induced by weak TBS alone, and whether it is age dependent or not.

**Fig. 5:**
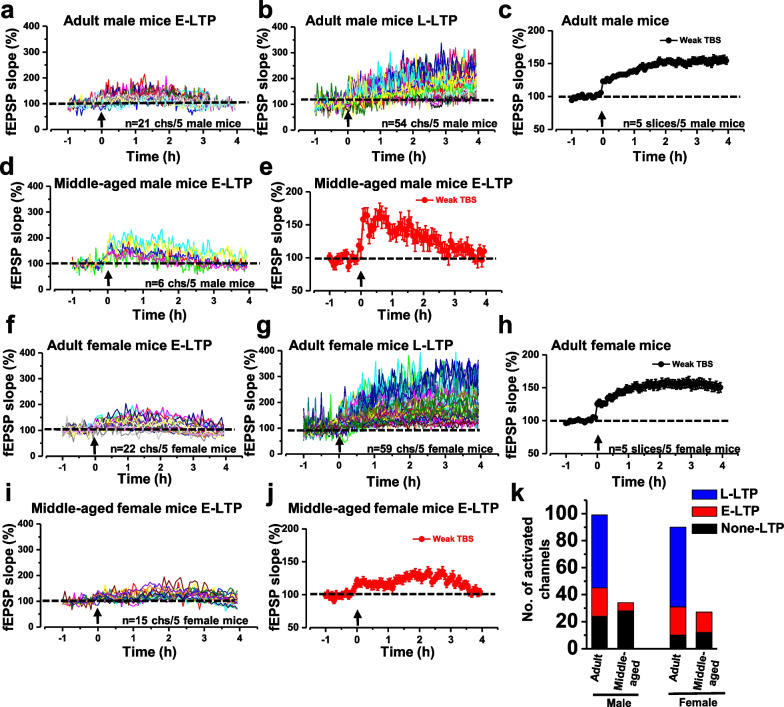
L-LTP was more easily induced by weak TBS in adult mice than in middle-aged mice. The induction of E-LTP (**a**) and L-LTP (**b**) in adult male mice. Summarized plot of the fEPSP slope demonstrates that weak TBS could induce L-LTP in adult male mice (n = 5 slices/5 mice) (**c**). The induction of E-LTP by weak TBS lasts for at least 2.5 h. Individual data of E-LTP (**d**) were induced by weak TBS in middle-aged male mice. Summarized plot of the fEPSP slope demonstrates that weak TBS could induce E-LTP in middle-aged male mice (n = 5 slices/5 mice) (**e**). The induction of E-LTP (**f**) and L-LTP (**g**) by weak TBS in adult female mice. Summarized plot of the fEPSP slope demonstrates that weak TBS could induce L-LTP in adult female mice (n = 5 slices/5 mice) (**h**). Individual data of E-LTP (**i**) were induced by weak TBS in middle-aged female mice. Summarized plot of the fEPSP slope demonstrates that weak TBS could induce E-LTP in middle-aged female mice (n = 5 slices/5 mice) (**j**). The bar plots are a quantitative representation of the number of activated channels induced by weak TBS in different ages and genders (**k**)

We attempted only delivering a single weak TBS, the same as tagging TBS (five trains of a burst with four 100 Hz pulses at 200 ms intervals), to the deep layer of ACC after obtaining a stable baseline for an hour. Interestingly, it was found that L-LTP could be induced by weak TBS in some channels in adult mice. However, there was no L-LTP induced by weak TBS in middle-aged mice (Fig. 5). In adult male mice, there were 99 channels activated in total after weak TBS, among which there were 21 channels showing E-LTP, 54 channels showing L-LTP and 24 channels showing non-LTP (n = 5 mice, Fig. 5a–c). The ratio of L-LTP channels was 54.55% in all activated channels (Fig. 5k). In middle-aged male mice, there were 34 channels activated, among which there were only 6 channels showing E-LTP and 28 channels showing non-LTP (n = 5 mice, Fig. 5d, e). No channel showed L-LTP in the middle-aged mice.

Similarly, in adult female mice, there were 91 channels activated, among which 22 channels were showing E-LTP, 59 channels showing L-LTP, and 10 channels showing non-LTP (n = 5 mice, Fig. 5f–h), and the ratio of L-LTP channels was 65.56% (Fig. 5k). In the middle-aged female mice, there were 27 channels activated, among which 15 channels were showing E-LTP and 12 channels showing non-LTP (Fig. 5i, j), and again no channel showed L-LTP. In summary, L-LTP can be induced by weak TBS in the ACC and shows an age-dependent manner in both male and female mice.

### BDNF rescued synaptic tagging LTP in middle-aged mice

**Fig. 6:**
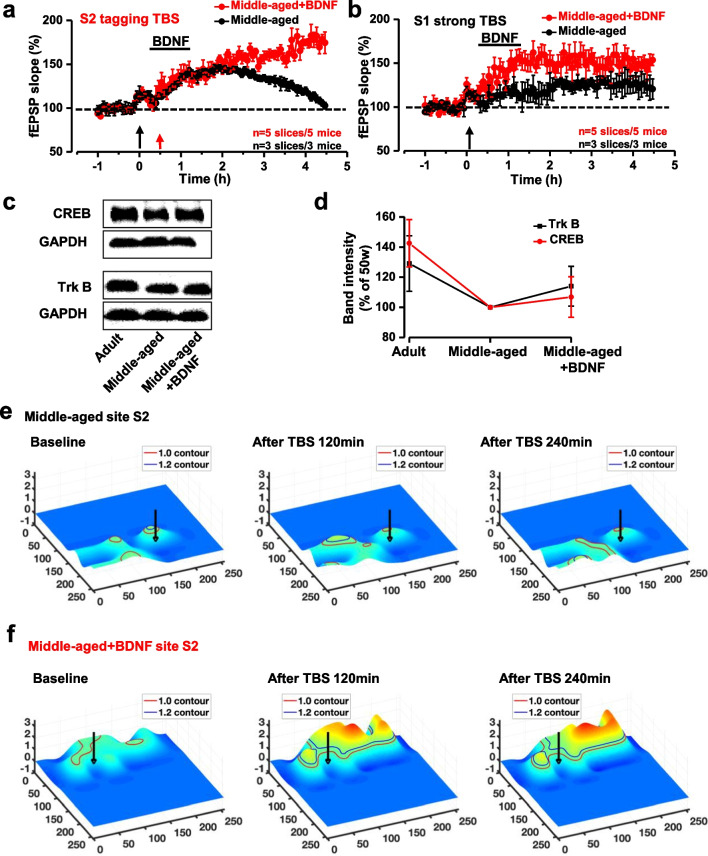
BDNF rescued the synaptic tagging in middle-aged mice. BDNF was applied in the brain slices of middle-aged male mice. Tagging LTP recorded around site S2 from the brain slices of the BDNF applied /unapplied in middle-aged male mice (**a**). LTP was recorded around site S1 from the brain slices of the same mice (**b**). Trk B, CREB levels were detected by western blot in three groups, adult mice, middle-aged mice, and BDNF-applied middle-aged mice (**c**). The intensity level of Trk B and CREB in each group, n = 5 for each group (**d**). The Markov Random Field model was introduced to smooth and visualize the low-resolution 8 × 8 MED64 data. The temporal evolving of the 3D surface on site S2 on the brain slice of the control middle-aged male mouse (**e**). The temporal evolving of the 3D surface on site S2 on the brain slice of the BDNF-incubated middle-aged male mouse (**f**)

Our previous study reported that BDNF contributes to synaptic potentiation in the ACC of adult mice (Miao et al. [36]). In this study, we tested whether BDNF can rescue the impaired tagging LTP in middle-aged mice. As shown in Fig. 6a, all the BDNF–incubated brain slices had synaptic tagging responses in middle-aged mice, and 35 channels (34.3%) exhibited clear synaptic tagging LTP. In control mice, only 2 brain slices from 5 middle-aged mice showed tagging-like response, and only 11 channels (9.73%) exhibited synaptic tagging LTP. In site S1 of strong TBS, it was also found that BDNF improved the L-LTP in the middle-aged mice (Fig. 6b). All the BDNF incubated brain slices had L-LTP after strong TBS, while only 3 brain slices from 5 mice showed L-LTP in no BDNF application slices.

In addition, the change of tyrosine kinase receptor B (Trk B) and cAMP-response element binding protein (CREB) were also tested in the middle-aged mice slices after the application of BDNF. The result from the western blot showed that both the Trk B and the CREB in the ACC increased after BDNF incubation in the middle-aged mice (Fig. 6c, d). These results are consistent with the previous reports [37, 38].

Next, we compared the Spatio-temporal distribution of the synaptic tagging LTP after BDNF in middle-aged mice by using the developed modeling and visualization system (Fig. 6e, f). After incubating BDNF, it is then observed that the adopted tagging-TBS exhibits multiple L-LTP peaks (vertices), representing the spatial strength and spatial frequency of the synaptic tagging changes in brain slices after the application of BDNF. This is consistent with our previous work [31] that the network LTP is often formed as a ring distribution surrounding the stimulation site.

### TrkB agonist R13 rescued synaptic tagging LTP in middle-aged mice

**Fig. 7:**
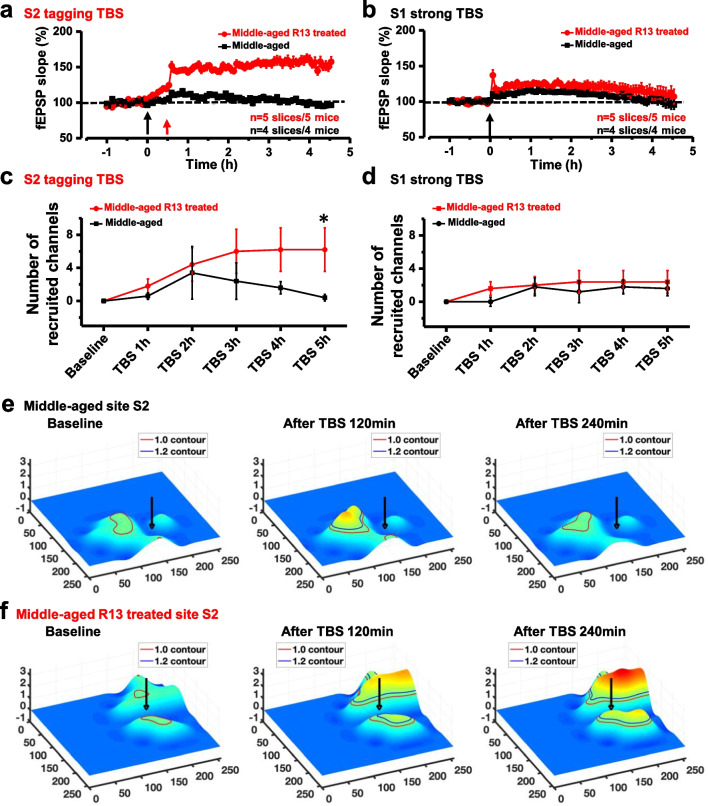
R13 rescued the synaptic tagging in middle-aged mice. Tagging was recorded around site S2 from the brain slices of the R13 treated/untreated middle-aged male mice (**a**). LTP around S1 was recorded around site S1 from the same brain slices (**b**). The temporal changes of the average number on sites S1 and S2 of the recruited channels in R13 treated and untreated mice. n = 5 slices from 5 R13 treated mice and n = 4 slices from 5 untreated mice, respectively (**c**, **d**). (*p < 0.05, n = 5 mice for each group, Student’s *t*-test.). The temporal evolving of the 3D surface on site S2 of the R13 untreated mouse brain slice (**e**). The temporal evolving of the 3D surface on site S2 of the R13 treatment mouse brain slice (**f**)

R13 is a prodrug for 7,8-dihydroxyflavone(7,8-DHF), which is a flavone found in plants and has a similar function to BDNF [39, 40]. We also tested the roles of R13 in the impaired tagging LTP in middle-aged mice. R13 was orally administered for 15 days in middle-aged mice, and then the brain slices were used for fEPSPs recording as above mentioned. As shown in Fig. 7, in R13-treated middle-aged mice, all the brain slices from 5 mice with R13 treatment had synaptic tagging response, while no brain slices from control mice showed tagging-like response (Fig. 7a). On site S1, we found that R13 enhanced the L-LTP in middle-aged mice (Fig. 7b). All the R13 treated mice shown L-LTP responses after strong TBS (n = 5 slices/ 5 mice), while only 4 mice showed E-LTP in control mice.

We also analyzed the recruited channels in R13-treated middle-aged mice. As shown in Fig. 7c, d, 4.5 h after strong TBS on site S1, 2.4 ± 1.4 channels were recruited in each slice with R13 treated mice, and 1.6 ± 0.9 channels in untreated mice(t_1h_ = 1.395, *p*_1h_ = 0.235 > 0.05; t_2h_ = 0.196, *p*_2h_ = 0.854 > 0.05; t_3h_ = 0.93, *p*_3h_ = 0.405 > 0.05; t_4h_ = 1, *p*_4h_ = 0.374 > 0.05; t_5h_ = 1.089, *p*_5h_ = 0.338 > 0.05, n = 5 mice for each group, Student’s t-test). After tagging TBS, 6.2 ± 2.6 channels were recruited in brain slices from R13 treated mice, and it was 0.4 ± 0.4 channels for the untreated mice (t_1h_ = 1.360, *p*_1h_ = 0.245 > 0.05; t_2h_ = 0.222, *p*_2h_ = 0.835 > 0.05; t_3h_ = 1.037, *p*_3h_ = 0.359 > 0.05; t_4h_ = 1.601, *p*_4h_ = 0.185 > 0.05; t_5h_ = 2.558, *p = 0.043 < 0.05, n = 5 mice for each group, Student’s t-test.). These results suggest that R13 enhances the network propagation of synaptic tagging responses in the ACC of middle-aged mice.

In addition, as revealed in Fig. 7e, f, where the Spatio-temporal distribution of synaptic plasticity response after R13 oral administration was visualized as a 3D surface. Taken together, these results show that the TrkB agonist rescued synaptic tagging and LTP in middle-aged mice.

## Discussion

The ACC is a critical forebrain structure involved in a variety of high-level brain functions, such as pain perception, memory storage, and emotional processing. Synaptic plasticity in the ACC has been proven to be the key cellular and synaptic substrate for chronic pain, fear, and anxiety [12, 41, 42]. In the present study, we report that synaptic tagging, hetero-synaptic plasticity, exists in adult excitatory synapses of the ACC. We found that synaptic tagging LTP in the ACC was impaired in both male and female middle-aged mice. The loss of synaptic tagging in mice indicates its relevance to the physiology changes in the brain of middle-aged mice. In addition, we found that TrkB agonists, both BDNF and R13, rescued the impaired synaptic tagging in middle-aged mice. Our study provides strong evidence that impaired cortical synaptic tagging may contribute to memory dysfunction in aged mice.

### Loss of synaptic tagging in middle-aged mice

Many studies have reported on the basic mechanisms and behavioral relevance of synaptic tagging in the hippocampus [43–46]. Synaptic tagging seeks to explain how neural signaling at a particular synapse creates a target for subsequent plasticity-related product (PRP) trafficking, which is essential for sustained LTP. Recently, our group found that synaptic tagging also occurred in the ACC [18]. Loss of synaptic tagging in ACC due to amputation may contribute to injury-related cognitive changes and phantom limb sensation and pain. It was also discovered that the spatial extent of tagging LTP propagation was greatly dependent on the interval time window. However, it has been unclear to this point if synaptic tagging correlates with aging.

In this study, we first demonstrated that synaptic tagging channels can perform in the ACC synapses in middle-aged mice. The synaptic tagging LTP was impaired in the ACC in middle-aged mice. Both the network L-LTP and the recruitment of inactive responses were reduced. As age increases, the ACC synapses may have some physiological change in response to stimulation, which leads to a change in synaptic tagging. Considering the importance of ACC synapses in pain perception, fear memory, and anxiety [2, 12, 47, 48], these findings suggest that synaptic tagging may have a more general physiological function in the brain. It is of great interest to further elucidate the molecular mechanisms of this cortical synaptic tagging and to dissect its behavioral relevance.

### No differences between gender in synaptic tagging

Gender differences in responses to brain diseases have been reported in both animal and human studies. In the present work, we find that there is no significant difference in synaptic tagging in the ACC between male and female mice. This is similar to findings in our previous work [49], which indicated that there is no gender-related difference in LTP in the ACC. However, in AD patients, some reports have shown that synaptic plasticity and associative memory impairments are more prominent in females due to the difference in hippocampus LTP [50]. These inconsistent results of gender differences in synaptic plasticity may be due to the different regions of the brain or various induction paradigms employed.

### L-LTP induced by weak TBS

Our previous studies found that weak TBS only induced declining E-LTP in the ACC for 2–3 h in adult mice [18]. We wonder whether weak TBS was able to induce L-LTP and whether it was age-dependent. However, it was found that L-LTP could be induced by weak TBS only in adult mice, but not (or much more rarely) in middle-aged mice. One possible explanation is that, younger mice have better synaptic plasticity that favors a lower fire threshold, while the induction protocols we used might be over strong and therefore biased over L-LTP. This result was also reported in previous work [51]. This result gives us a new way to define the strong effect of the TBS protocol.

### TrkB agonist rescued synaptic tagging in middle-aged mice

BDNF plays a diverse, and broad, role in regulating neuronal structure and function in the central nervous system [52]. Our previous study reported that BDNF can contribute to synaptic potentiation in the ACC of adult mice (Miao et al. [36]). In present studies, we adopt BDNF to explore whether it can contribute to synaptic tagging in the ACC or not. We found that BDNF can rescue the impaired synaptic tagging and network LTP in middle-aged mice. Previous studies reported that 7,8-DHF can imitate the function of BDNF [39, 40]. R13, as an optimal prodrug of 7,8-DHF, increases the half-life, oral bioavailability, and brain exposure of 7,8-DHF [53]. We found that similar to BDNF, R13 also revised the impaired synaptic tagging and network LTP in middle-aged mice. These results showed that R13 may be a potential therapeutic agent for the treatment of memory loss, which is consistent with the previous reports [53].

### BDNF-TrkB signaling in middle-aged mice

In the present study, we found that BDNF and a TrkB receptor agonist R13 both rescued aged-related reduction of synaptic tagging. These results indicate that BDNF-related signaling pathway maybe altered during aging. In consistent with this observation, reduced BDNF levels and altered BDNF-TrkB signaling have been reported in aged mice. For example, total BDNF mRNA and the expression level of TrkB receptor were reduced in 12-month-old rat hippocampus [54]. It is known that BDNF is expressed in the hippocampus and cortex in the brain, and can be synthesized by both neurons and glial cells. While a previous report showed that BDNF gene expression in microglia is at very low level in the adult brain [55], Zhou et al. reported that microglial BDNF deletion prevents high-frequency stimulation-induced LTP [56]. Future studies are clearly needed to determine the exact cellular mechanism for BDNF and its release in the ACC of both adult and aged animals.

In the present study, it is quite likely that BDNF-TrkB signaling contributes to the rescuing of synaptic tagging by regulating the AMPAR expression and function, including synaptic AMPAR subunit trafficking and phosphorylation. Our previous study has found that selective phosphorylation of AMPAR at the PKA phosphorylation site serine 845 contributes to the network of LTP expression in the ACC [31]. Furthermore, Miao et al. found that BDNF-induced enhancement in the ACC is dependent on L-VGCC, calcium-stimulated adenylyl cyclase subtype 1(AC1) and postsynaptic incorporation of calcium-permeable AMPA receptors (CP-AMPARs) [36]. In the hippocampus, it has also been reported that BDNF-TrkB signaling enhances synaptic transmission by upregulating the AMPAR subunit trafficking on the postsynaptic membrane [57, 58].

### The novel fEPSP signal modeling and visualization system

In our research, by using the MED64 system, the LTP could be recorded from up to 64 channels for one ACC slice, as compared to the traditional methods that are based on two-electrode (one for stimulation and one for recording). Unfortunately, to obtain a more comprehensive understanding of the Spatio-temporal dynamics of signals and responses in a real network, the number of available activations is only 8 × 8 which is still insufficient. To overcome the constraint, we consider the original MED64 recording only as a sparse observation from a latent state space that could be either discrete or continuous, and by estimating the values of the state variables, we can easily project them back into a smoother observation space such that visualizing and analyzing the Spatio-temporal dynamics of activation and response becomes easier. In our previous work, we attempted modeling such a process for a single time frame with a Gaussian Mixture Model [31]. In this paper, we generalize the problem with a state space model (“A novel fEPSP signal modeling and visualization system” section), and by adopting the graph theory, we can build a Markov Random Field based on the MED64 observation to estimate the latent state variable, and then project the original 8 × 8 input into a higher resolution output (256 in our experiments). Such an observation-state-projection pipeline is generic enough to take observations from both single time frame (the spatial case) as well as multiple time frames (the Spatio-temporal case), and can also project into a single higher resolution estimation (this paper) as well as multiple higher resolution estimations (on-going work). In fact, we believe that our proposed model aligns well with the unknown mechanism of ACC where the recorded MED64 signal (the observation) depends on the behavior of the ACC neurons (the state), and once we obtain an accurate estimation of the state value, we can further estimate responses at a finer granularity (the high-resolution projection). Such a generic model could apply to many other similar scenarios.

In summary, our results demonstrate that middle-aged mice have less ability to induce the synaptic tagging and recruitment of cortical circuitry in the ACC. Application of TrkB agonist can rescue the synaptic tagging in middle-aged mice. Since L-LTP is important for the process of anxiety and fear memory, further studies should investigate the detection of the relationship between strong TBS site and tagging TBS site in synaptic tagging, as well as an expanded application such as the possible changed spatial–temporal properties of network L-LTP in anxiety and fear memory.

## Data Availability

All data generated or analyzed during this study are included in this published article.

## References

[1] Bliss and Collingridge (2013). Expression of NMDA receptor-dependent LTP in the hippocampus: bridging;the divide. J Molecular Brain.

[2] Bliss TV (2016). Synaptic plasticity in the anterior cingulate cortex in acute and chronic pain. Nat Rev Neurosci.

[3] Asok A (2019). Molecular mechanisms of the memory trace. Trends Neurosci.

[4] Chen T (2014). Adenylyl cyclase subtype 1 is essential for late-phase long term potentiation and spatial propagation of synaptic responses in the anterior cingulate cortex of adult mice. Mol Pain.

[5] Park P (2016). Calcium-permeable AMPA receptors mediate the induction of the protein kinase a-dependent component of long-term potentiation in the hippocampus. J Neurosci.

[6] Li XY (2010). Alleviating neuropathic pain hypersensitivity by inhibiting PKMzeta in the anterior cingulate cortex. Science.

[7] Frey U, Morris RG (1997). Synaptic tagging and long-term potentiation. Nature.

[8] Frey U, Morris RGM (1998). Synaptic tagging: implications for late maintenance of hippocampal long-term potentiation. Trends Neurosci.

[9] Reymann KG, Frey JU (2007). The late maintenance of hippocampal LTP: requirements, phases, 'synaptic tagging', 'late-associativity' and implications. Neuropharmacology.

[10] Park P (2019). On the Role of Calcium-Permeable AMPARs in Long-Term Potentiation and Synaptic Tagging in the Rodent Hippocampus. Front Synaptic Neurosci.

[11] Zhuo M (2008). Cortical excitation and chronic pain. Trends Neurosci.

[12] Zhuo M (2016). Neural Mechanisms Underlying Anxiety-Chronic Pain Interactions. Trends Neurosci.

[13] Vogt BA (2005). Pain and emotion interactions in subregions of the cingulate gyrus. Nat Rev Neurosci.

[14] Tang J (2005). Pavlovian fear memory induced by activation in the anterior cingulate cortex. Mol Pain.

[15] Vetere G (2011). Spine growth in the anterior cingulate cortex is necessary for the consolidation of contextual fear memory. Proc Natl Acad Sci U S A.

[16] Frankland PW, Teixeira CM (2005). A pain in the ACC. Mol Pain.

[17] Tanimizu T (2017). Functional connectivity of multiple brain regions required for the consolidation of social recognition memory. J Neurosci.

[18] Liu MG, Song Q, Zhuo M (2018). Loss of synaptic tagging in the anterior cingulate cortex after tail amputation in adult mice. J Neurosci.

[19] Rogerson T (2014). Synaptic tagging during memory allocation. Nat Rev Neurosci.

[20] Kastellakis G (2015). Synaptic clustering within dendrites: an emerging theory of memory formation. Prog Neurobiol.

[21] Luboeinski J, Tetzlaff C (2021). Memory consolidation and improvement by synaptic tagging and capture in recurrent neural networks. Commun Biol.

[22] Bergado JA, Lucas M, Richter-Levin G (2011). Emotional tagging–a simple hypothesis in a complex reality. Prog Neurobiol.

[23] Vecsey CG, Huang T, Abel T (2018). Sleep deprivation impairs synaptic tagging in mouse hippocampal slices. Neurobiol Learn Mem.

[24] Bach ME (1999). Age-related defects in spatial memory are correlated with defects in the late phase of hippocampal long-term potentiation in vitro and are attenuated by drugs that enhance the cAMP signaling pathway. Proc Natl Acad Sci U S A.

[25] Wimmer ME (2012). Aging impairs hippocampus-dependent long-term memory for object location in mice. Neurobiol Aging.

[26] Shoji H (2016). Age-related changes in behavior in C57BL/6J mice from young adulthood to middle age. Mol Brain.

[27] Wong LW (2021). Age-related changes in hippocampal-dependent synaptic plasticity and memory mediated by p75 neurotrophin receptor. Aging Cell.

[28] Gros A, Wang SH (2018). Behavioral tagging and capture: long-term memory decline in middle-aged rats. Neurobiol Aging.

[29] Bramham CR, Messaoudi E (2005). BDNF function in adult synaptic plasticity: the synaptic consolidation hypothesis. Prog Neurobiol.

[30] Xue M (2021). NMDA receptor-dependent synaptic depression in potentiated synapses of the anterior cingulate cortex of adult mice. Mol Pain.

[31] Song Q (2017). Selective phosphorylation of AMPA receptor contributes to the network of long-term potentiation in the anterior cingulate cortex. J Neurosci.

[32] Li XH (2021). Oxytocin in the anterior cingulate cortex attenuates neuropathic pain and emotional anxiety by inhibiting presynaptic long-term potentiation. Cell Rep.

[33] Kindermann R, Snell JL (1980). Markov random fields and their applications.

[34] Turkowski K (1990). Filters for common resampling tasks.

[35] Chen T (2014). Pharmacological rescue of cortical synaptic and network potentiation in a mouse model for fragile X syndrome. Neuropsychopharmacology.

[36] Miao HH (2021). Brain-derived neurotrophic factor produced long-term synaptic enhancement in the anterior cingulate cortex of adult mice. Mol Brain.

[37] Caldeira MV (2007). BDNF regulates the expression and traffic of NMDA receptors in cultured hippocampal neurons. Mol Cell Neurosci.

[38] Leal G, Comprido D, Duarte CB (2014). BDNF-induced local protein synthesis and synaptic plasticity. Neuropharmacology.

[39] Wurzelmann M, Romeika J, Sun D (2017). Therapeutic potential of brain-derived neurotrophic factor (BDNF) and a small molecular mimics of BDNF for traumatic brain injury. Neural Regen Res.

[40] Liu C, Chan CB, Ye K (2016). 7,8-dihydroxyflavone, a small molecular TrkB agonist, is useful for treating various BDNF-implicated human disorders. Transl Neurodegener.

[41] Zhao MG (2005). Roles of NMDA NR2B subtype receptor in prefrontal long-term potentiation and contextual fear memory. Neuron.

[42] Koga K (2015). Coexistence of two forms of LTP in ACC provides a synaptic mechanism for the interactions between anxiety and chronic pain. Neuron.

[43] Young JZ (2006). Metaplasticity of the late-phase of long-term potentiation: a critical role for protein kinase A in synaptic tagging. Eur J Neurosci.

[44] Ramachandran B, Frey JU (2009). Interfering with the actin network and its effect on long-term potentiation and synaptic tagging in hippocampal CA1 neurons in slices in vitro. J Neurosci.

[45] Sajikumar S, Korte M (2011). Metaplasticity governs compartmentalization of synaptic tagging and capture through brain-derived neurotrophic factor (BDNF) and protein kinase Mzeta (PKMzeta). Proc Natl Acad Sci U S A.

[46] Dasgupta A (2017). Substance P induces plasticity and synaptic tagging/capture in rat hippocampal area CA2. Proc Natl Acad Sci U S A.

[47] Keum S (2018). A Missense variant at the Nrxn3 locus enhances empathy fear in the mouse. Neuron.

[48] Sellmeijer J (2018). Hyperactivity of anterior cingulate cortex areas 24a/24b drives chronic pain-induced anxiodepressive-like consequences. J Neurosci.

[49] Liu RH (2020). Sex difference in synaptic plasticity in the anterior cingulate cortex of adult mice. Mol Brain.

[50] Navakkode S (2021). Sex-specific accelerated decay in time/activity-dependent plasticity and associative memory in an animal model of Alzheimer's disease. Aging Cell.

[51] Radulescu CI (2021). The aging mouse brain: cognition, connectivity and calcium. Cell Calcium.

[52] Panja D, Bramham CR (2014). BDNF mechanisms in late LTP formation: a synthesis and breakdown. Neuropharmacology.

[53] Chen C (2018). The prodrug of 7,8-dihydroxyflavone development and therapeutic efficacy for treating Alzheimer's disease. Proc Natl Acad Sci U S A.

[54] Perovic M (2013). BDNF transcripts, proBDNF and proNGF, in the cortex and hippocampus throughout the life span of the rat. Age (Dordr).

[55] Bennett ML (2016). New tools for studying microglia in the mouse and human CNS. Proc Natl Acad Sci U S A.

[56] Zhou LJ (2019). Microglia are indispensable for synaptic plasticity in the spinal dorsal horn and chronic pain. Cell Rep.

[57] Zhang H (2018). Modulation of AMPA receptor surface diffusion restores hippocampal plasticity and memory in Huntington's disease models. Nat Commun.

[58] Keifer J (2022). Regulation of AMPAR trafficking in synaptic plasticity by BDNF and the impact of neurodegenerative disease. J Neurosci Res.

